# Four-Dimensional Computed Tomography Based Respiratory-Gated Radiotherapy with Respiratory Guidance System: Analysis of Respiratory Signals and Dosimetric Comparison

**DOI:** 10.1155/2014/306021

**Published:** 2014-09-07

**Authors:** Jung Ae Lee, Chul Yong Kim, Dae Sik Yang, Won Sup Yoon, Young Je Park, Suk Lee, Young Bum Kim

**Affiliations:** Department of Radiation Oncology, Korea University College of Medicine, Korea University Medical Center, 126-1 Anamdong 5ga, Seongbukgu, Seoul 136-705, Republic of Korea

## Abstract

*Purpose*. To investigate the effectiveness of respiratory guidance system in 4-dimensional computed tomography (4DCT) based respiratory-gated radiation therapy (RGRT) by comparing respiratory signals and dosimetric analysis of treatment plans. *Methods*. The respiratory amplitude and period of the free, the audio device-guided, and the complex system-guided breathing were evaluated in eleven patients with lung or liver cancers. The dosimetric parameters were assessed by comparing free breathing CT plan and 4DCT-based 30–70% maximal intensity projection (MIP) plan. *Results*. The use of complex system-guided breathing showed significantly less variation in respiratory amplitude and period compared to the free or audio-guided breathing regarding the root mean square errors (RMSE) of full inspiration (*P* = 0.031), full expiration (*P* = 0.007), and period (*P* = 0.007). The dosimetric parameters including *V*_5 Gy_, *V*_10 Gy_, *V*_20 Gy_, *V*_30 Gy_, *V*_40 Gy_, and *V*_50 Gy_ of normal liver or lung in 4DCT MIP plan were superior over free breathing CT plan. *Conclusions*. The reproducibility and regularity of respiratory amplitude and period were significantly improved with the complex system-guided breathing compared to the free or the audio-guided breathing. In addition, the treatment plan based on the 4D CT-based MIP images acquired with the complex system guided breathing showed better normal tissue sparing than that on the free breathing CT.

## 1. Introduction

**Figure 1:**
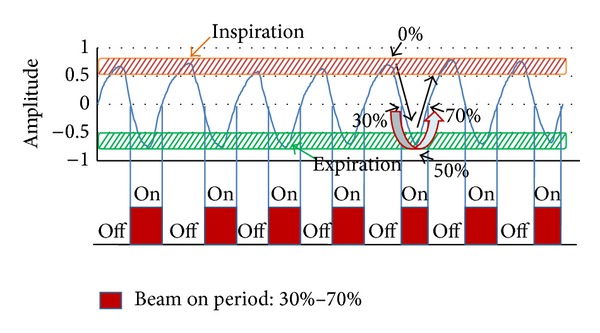
An example of the respiratory gating radiation therapy during the specific period of respiration.

Radiation therapy (RT) has evolved with the state-of-the-art technology with the purpose of giving higher doses to the tumors and lower doses to the surrounding normal tissues to maximize tumor control and minimize toxicities. The four-dimensional RT (4DRT), adding the fourth element “time” to the three-dimensional conformal RT (3DCRT), has been introduced and actively performed in daily practice in the same vein. Organs in the thorax and the abdomen are mostly affected by the respiratory motion moving up to 10–40 mm according to the literature [1, 2]. Respiration is quasiperiodic with a relatively predictable pattern in contrast to the arbitrary changes including peristalsis, expansion, contraction, and incidental cough which are difficult to predict [3]. The respiration-gated RT (RGRT) using the 4DRT technique permits selectively treating specific respiratory phases in the light of target location according to the organ movement and increasing therapeutic efficiency [4, 5]. Larger fields with larger safety margins to the target volumes were generally used to overcome the uncertainties during the treatment in the past [6]. However, targets have been getting smaller and more conformal with precise tumor localization for increasing tumor control and reducing the risk of toxicities. There have been several techniques to reduce the margins caused by the respiratory motion. These are breath holding techniques including active breathing control (ABC), abdomen compression, deep inspiration breathing hold technique (DIBH), respiratory gating technique, real-time tumor-tracking RT (RTRT), multileaf collimator (MLC) sequencing technique, couch tracking technique, and cyberknife robot RT [7–14]. Of these, gating technique denotes “beam-on” during the specific respiration phases and “beam-off” during the other phases as shown in Figure 1. To ensure the regularity and reproducibility of respiration during the RGRT, the audio-guided or the video-guided feedback techniques have been suggested and shown to be beneficial when compared to the free breathing [15, 16]. The aim of this study was to investigate the effectiveness of respiratory guidance system including audio and visual devices in 4-dimensional computed tomography (4DCT) based RGRT by comparing respiratory signals of free breathing (no feedback), audio-guided breathing, and complex system-guided breathing including audio and visual feedback.

## 2. Materials and Methods

### 2.1. Patients

Eleven patients were enrolled in an Institutional Review Board-approved respiratory guidance system protocol for 4D RGRT from March 2011 to July 2013 in Korea University Guro Hospital. The inclusion criteria were that the patient (1) was diagnosed as a tissue-confirmed liver or lung cancer; (2) was over 18 years of age; (3) was not oxygen-dependent; (4) has Eastern Cooperative Oncology Group Performance Status (ECOG PS) of 0–2; (5) showed the diaphragmatic or visible tumor movement of more than 1 cm in a craniocaudal direction in a X-ray fluoroscopy; (6) had given signed informed consent.

### 2.2. The Respiratory Guidance System

**Figure 2:**
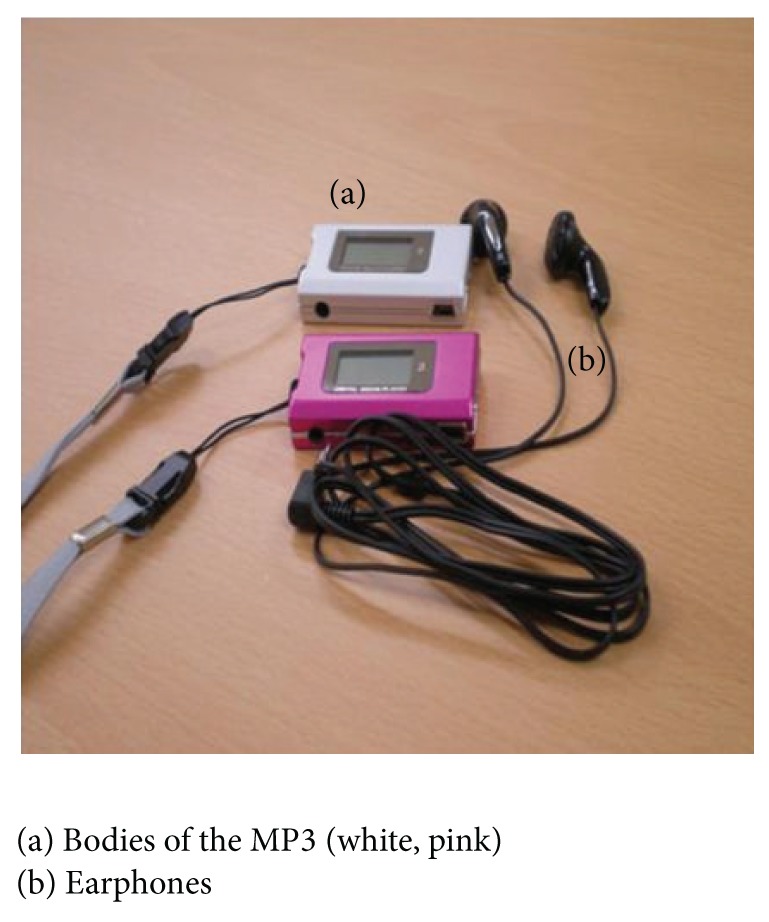
The audio device of the respiratory guidance system.

**Figure 3:**
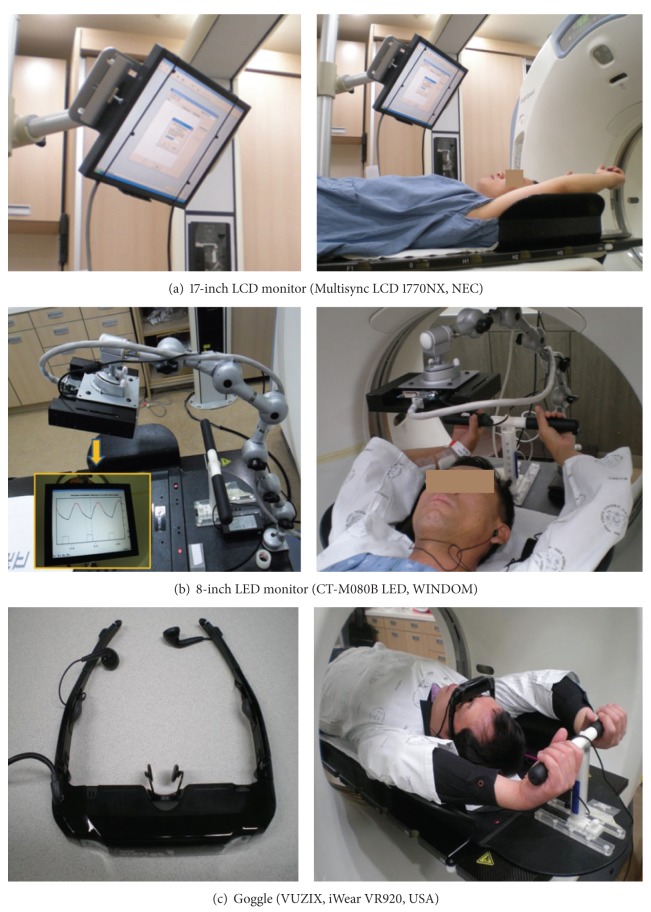
The visual devices of the respiratory guidance system.

The respiratory guidance system reflecting the real-time breathing status for the 4DCT and the RGRT consisted of two parts: the audio-component and the visual component. The audio-component was composed of the motion picture expert group 1 audio layer 3 (MP3) and earphones installed with voice files guiding the patient's respiration from 10 to 22 times per minute which were recorded according to the individual speed of breathing (Figure 2). For the visual components, the patients were able to choose one of the three types of visual devices showing the patient's real time breathing signals: 17-inch liquid crystal display (LCD) monitor (Multisync LCD 1770NX, NEC), 8-inch light emitting diode (LED) monitor (CT-M080B LED, WINDOM), or goggle (VUZIX, iWear VR920, USA). The monitor types were installed to the couch by monitor arm with six joints and were preferred for the patients wearing glasses (Figure 3).

### 2.3. Acquisition of Respiratory Signals

**Figure 4:**
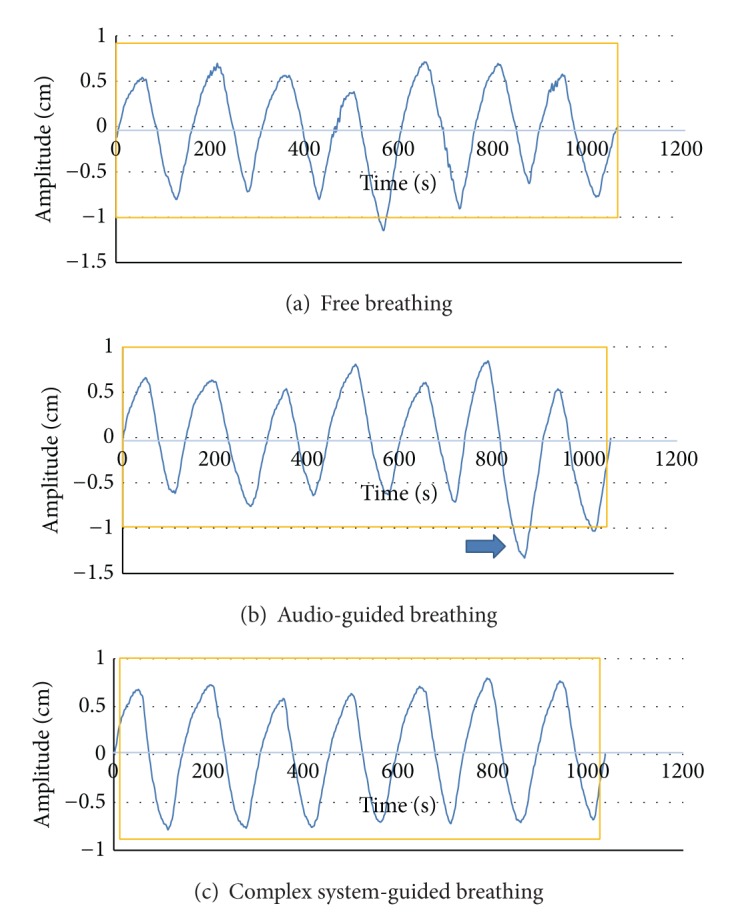
Examples of the free breathing, the audio-guided breathing, and the complex system-guided breathing (the arrow indicates a deviation caused by a transient positional change).

**Figure 5:**
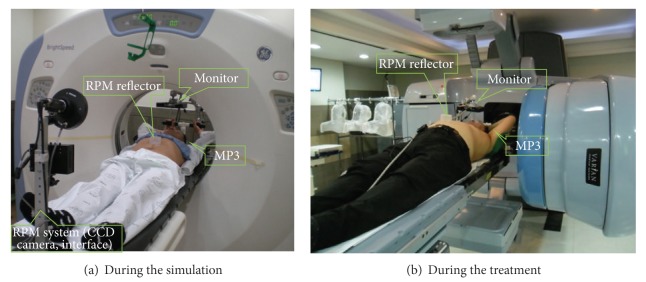
Patient setup with the complex system-guided respiration.

The individual patient's respiration was assessed by a separate session for respiration training to determine the best fitted speed of breathing of the audio instruction. The free breathing (no feedback), the audio-guided breathing, and the complex system-guided breathing including audio and visual feedback were applied to the patients and the respective respiration signals were collected for 5 minutes during the separate respiration training session and during the simulation (Figure 4). We used the Real Time Position Management (RPM) system (Varian Medical Systems, Palo Alto, CA) to acquire the patients' respiratory traces during the training session and the 4DCT simulation. The RPM system is composed of infrared-reflecting marker box which is placed on the patient's chest wall near xyphoid process and a charge coupled device (CCD) camera tracking the vertical motion of the marker at a frequency of 30 frames per second. The data collection and visualization are managed by a dedicated software tool installed in the system. The signals of the free breathing were acquired without any feedback. Then, the signals of the audio-guided breathing were obtained with breathe-in/breathe-out instructions. Finally the traces from the complex system-guided breathing were collected with the audio instruction as well as the real time visual feedback displayed in the selected monitor or goggle (Figure 5).

### 2.4. Analysis of the Respiratory Signals

**Figure 6:**
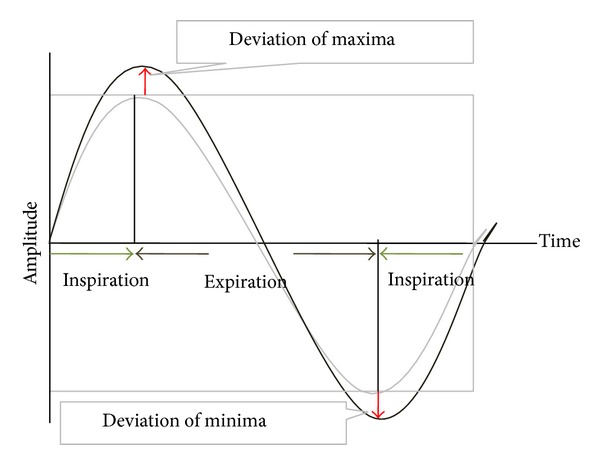
The analysis of the respiratory signals.

To evaluate the reproducibility and regularity of the respiration, data of the first and the last 15 seconds were excluded from the analysis regarding the adaptation period for the beginning period and time-related fatigue of the endmost period. The extreme outlier was also ruled out to increase the reliability of the results. The average of the three consecutive stable respiratory cycles was set up as a reference and normalized to “0” for the purpose of correlating signals from the different sessions and patients. The average of the maximal inspiration (maxima), the maximal expiration (minima), and the period of the reference respiratory cycles were set as a standard, and the respective root mean square errors (RMSE) were calculated for the entire signals (Figure 6).

### 2.5. Acquisition of Simulation CT and Analysis and Comparison of Treatment Plans

Two sets of simulation CT were performed in each patient using BrightSpeed Elite CT (GE healthcare Technologies, Waukesha, WI): first acquisition of precontrast enhanced CT under free breathing condition and then second acquisition of intravenous contrast agent enhanced 4DCT with complex system-guided breathing. The slice thickness was 5 mm. For the purpose of data reconstruction of 4DCT, the acquired images were sorted by 10 respiratory phases determined from the RPM system. The gating ranges were set between 30% and 70% phases, and images of 30%, 40%, 50% 60%, and 70% phases were reconstructed with maximum intensity projection (MIP) method. The datasets were imported to Eclipse planning system (Version 8.3, Varian, CA, USA) for contouring target volumes and organs at risk (OARs). The gross tumor volumes (GTVs) in both free breathing CT images and 4DCT based MIP images were delineated in each patient by the same radiation oncologist with appropriate window setting for lung and liver cancers, respectively. The clinical target volumes (CTVs) were created by adding 5 mm margin to the GTVs and additional 5 mm margins to the CTVs were allowed for the PTVs. To assess the dosimetric impacts on normal lung and liver, identical beam arrangements were generated in both free breathing CT images and 4DCT based MIP images. All of the plans used 3-4 coplanar beams and were normalized such that at least 95% of the PTV received the prescription dose. The treatment plans based on the free breathing CT were generated with the PTVs plus 1.5 cm margins and those on the 4DCT based MIP images were with the PTVs plus 1.0 cm since the internal margin of 5 mm was already reflected in the 4D simulation process. The dose fractionation schedules were 50 Gy in 5 fractions for the patients with lung cancer and 54 Gy in 18 fractions for those with liver cancer. The conformity index (CI), homogeneity index (HI), and prescription isodose target volume conformal index (PITV) were calculated for the comparison of target coverage [17–19]. For the DVH comparison of normal lung and liver, *V*_5 Gy_ (volume receiving 5 Gy), *V*_10 Gy_ (volume receiving 10 Gy), *V*_20 Gy_ (volume receiving 20 Gy), *V*_30 Gy_ (volume receiving 30 Gy), *V*_40 Gy_ (volume receiving 40 Gy), and *V*_50 Gy_ (volume receiving 50 Gy) were used. The actual treatment was done with gating 4DRT ranging between 30% and 70% using Trilogy (Varian Medical Systems Palo Alto, CA). The Kolmogorov-Smirnov test was done for the continuous variables. The paired Student's *t*-test and one way ANOVA in SPSS version 20 (SPSS Inc., Chicago, IL) were used for the statistical analysis. The *P* value of 0.05 or less was considered as statistically significant.

## 3. Results

**Figure 7:**
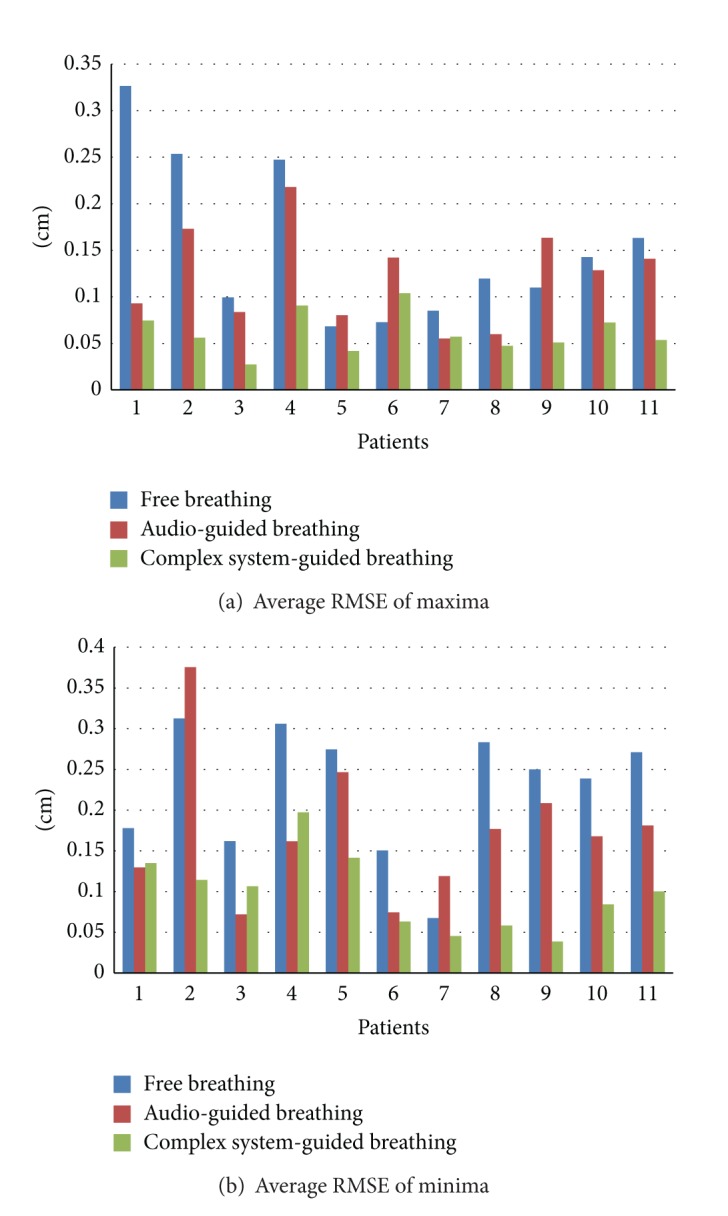
Root mean square error (RMSE) of the maxima and minima in all patients.

The patients' characteristics were listed in Table 1. Among the enrolled eleven patients, 6 patients had hepatocellular carcinoma and 5 had non-small-cell lung cancer. The median age was 64 years (range 52–80 years). No patient had a surgery of liver or lung except 2 with liver cancer who underwent partial hepatectomy. The concurrent chemotherapy was not done in all patients. The average respiration per minute was 14.6 times ± 2.8 and the average craniocaudal movement of diaphragm or tumor was 1.91 cm ± 0.70 in a routine fluoroscopy. The RMSEs of the maxima, the minima, and the period according to the three respiratory conditions were summarized in Table 2.  Figure 7 presented the RMSE of the maxima and minima of individual patient. The RMSE of the maxima indicating full inspiration was 0.154 cm ± 0.096 for the free breathing, 0.119 cm ± 0.057 for the audio-guided breathing, and 0.061 cm ± 0.024 for the complex system-guided breathing (*P* < 0.05). When comparing the RMSE of the maxima between two groups, there was no significant difference observed between the free and the audio-guided breathing (*P* = 0.216). However, statistical significance was presented between the free and the complex system-guided breathing (*P* = 0.004) and between the audio-guided and the complex system-guided breathing (*P* = 0.001). The RMSE of the minima indicating full expiration was 0.220 cm ± 0.085 for the free breathing, 0.174 cm ± 0.095 for the audio-guided breathing, and 0.099 cm ± 0.053 for the complex system-guided breathing (*P* < 0.05). Two group comparisons in the RMSE of the minima showed significant differences for all combinations: the free versus the audio-guided breathing, *P* = 0.020; the free versus the complex system-guided breathing, *P* = 0.000; the audio-guided versus the complex system-guided breathing, *P* = 0.020, respectively. Meanwhile, the RMSE of the period was 0.951 sec ± 0.130 for the free breathing, 0.741 sec ± 0.213 for the audio-guided breathing, and 0.464 sec ± 0.215 for the complex system-guided breathing (*P* < 0.05). There was no difference observed between the free and the audio-guided breathing (*P* = 0.370), but the differences in the RMSE of the period between the free and the complex system-guided breathing (*P* = 0.009) and between the audio-guided and the complex system-guided breathing (*P* = 0.040) were significant.

With the positive results, the 4DRT with complex system-guided breathing was applied to the actual treatment, and comparison between free breathing and 4DRT with complex system-guided breathing was performed in terms of dosimetric parameters. The CI, HI, and PITV were evaluated for comparison of target coverage between plans of free breathing CT and 4D MIP CT. The CI of free breathing CT was 0.578 ± 0.525 and that of 4D MIP CT was 0.637 ± 0.967, respectively (*P* = 0.096). The HI was 1.043 ± 0.024 and 1.033 ± 0.018 for free breathing CT and 4D MIP CT, respectively (*P* = 0.151). Likewise, the PITV of free breathing CT was 0.997 ± 0.007 and that of 4D MIP CT was 0.998 ± 0.007, respectively (*P* = 0.335). There were no significant differences observed between the plans in terms of target coverage.

**Figure 8:**
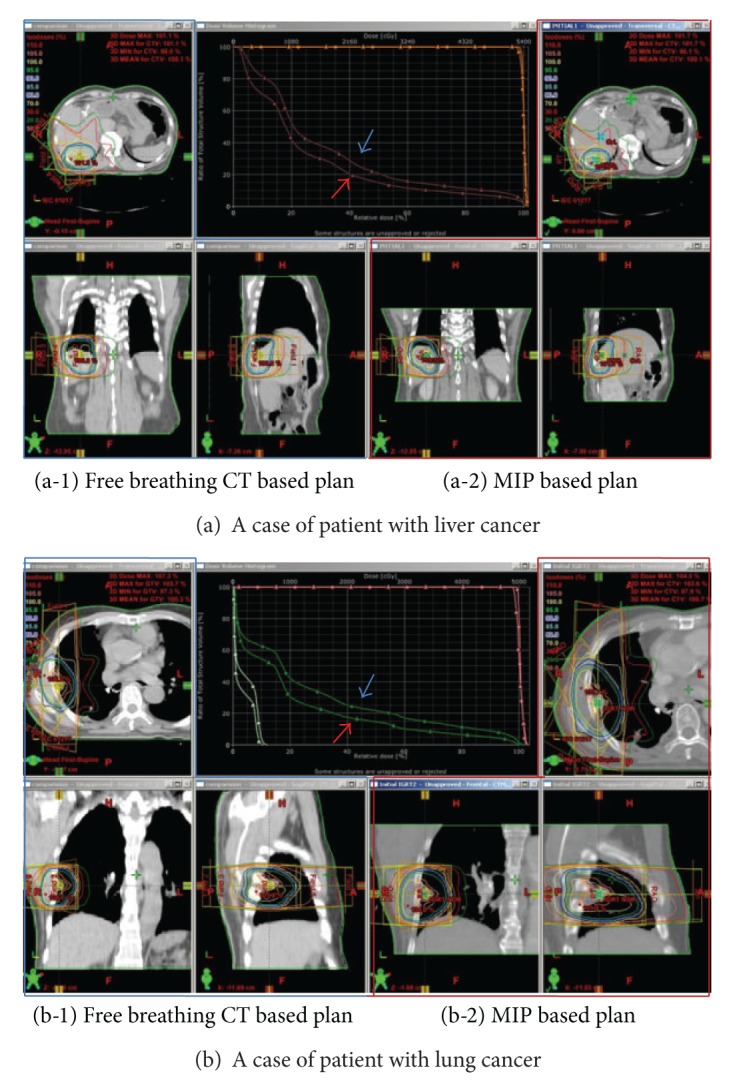
Comparison of dose volume histogram (DVH) of (a) liver and (b) lung between plans with free breathing CT (blue box and blue arrow) and 4D MIP CT (red box and red arrow) was shown.

The DVH analysis was done for liver and lung for each applicable site (Table 3 and Figure 8). Normal liver volume receiving the same dose of radiation was less in plan on 4D MIP CT than on free breathing CT in patients with liver cancer: 78.75 ± 3.51 cc in free breathing CT versus 68.06 ± 6.68 cc in 4D MIP CT (*P* = 0.024) for *V*_5 Gy_, 65.16 ± 7.83 cc versus 54.24 ± 10.45 cc (*P* = 0.030) for *V*_10 Gy_, 43.84 ± 12.80 cc versus 35.22 ± 14.11 cc (*P* = 0.048) for *V*_20 Gy_, 32.81 ± 12.68 cc versus 27.34 ± 14.68 cc (*P* = 0.117) for *V*_30Gy_, 25.79 ± 11.47 cc versus 21.53 ± 13.61 cc (*P* = 0.069) for *V*_40 Gy_, and 19.39 ± 9.71 cc versus 16.06 ± 11.21 cc (*P* = 0.067) for *V*_50 Gy_, respectively, as shown in Table 3(a). *V*_5 Gy_, *V*_10 Gy_, and *V*_20 Gy_ showed statistically significant differences. Ipsilateral lung volume receiving same dose of radiation for patients with lung cancer showed similar results (Table 3(b)). There were significant differences observed in *V*_10 Gy_, *V*_20 Gy_, *V*_30 Gy_, and *V*_40 Gy_ and *V*_5 Gy_ showed marginal significance: 48.97 ± 15.37 cc in free breathing CT versus 40.74 ± 11.07 cc in 4D MIP CT (*P* = 0.051) for *V*_5 Gy_, 37.71 ± 17.89 cc versus 29.31 ± 15.22 cc (*P* = 0.035) for *V*_10 Gy_, 23.02 ± 11. c versus 16.03 ± 8.75 cc (*P* = 0.018) for *V*_20Gy_, 15.19 ± 9.24 cc versus 8.94 ± 5.46 cc (*P* = 0.034) for *V*_30 Gy_, 9.18 ± 5.88 cc versus 4.90 ± 3.18 cc (*P* = 0.035) for *V*_40 Gy_, and 1.64 ± 1.93 cc versus 1.19 ± 1.35 cc (*P* = 0.287) for *V*_50 Gy_, respectively. There were no significant differences in *V*_5 Gy_ of contralateral lung volume (13.65 ± 12.29 cc in free breathing CT versus 8.91 ± 20.58 cc in 4D MIP CT, *P* = 0.140) and *V*_10 Gy_ (2.63 ± 4.90 cc versus 1.01 ± 2.08 cc, *P* = 0.160) as in Table 3(c).

## 4. Discussion

The “reproducibility” and “regularity” of respiration are important elements for treating moving tumors influenced by breathing in the 4D era. The RGRT with undefined reproducibility or regularity may result in the decrease of tumor dose and unintentional increase in the surrounding normal tissue. Therefore, it is vital to induce the patient's respiration as regularly as possible during the 4D RGRT. In addition, even breathing is also important for getting quality-guaranteed 4D images with the 4DCT. Various methods to reduce the artifact of the 4DCT and improve quality of the RGRT were introduced during the last ten-year period. As one aspect of it, the audio instruction or the video-guided respiration was reported in several groups of USA and Europe. Kini et al. [15] reported that the RGRT with respiratory training showed improved reproducibility compared to the RGRT without training. Studies comparing the free breathing and the audio-guided breathing revealed that audio instruction provides stable respiratory pattern compared to the free breathing in a limited extent [20, 21]. In addition, Cerviño et al. [22] reported that the visual feedback showed significant benefit to stabilize the respiration for the patients with breast cancer compared to the free breathing. Recent researches on the RGRT using both the audio and the visual guidance demonstrated more meaningful results. The audiovisual feedback system significantly reduced the breathing motion compared to the free or the audio-guided breathing according to George et al. [16]. Crossmann [23] and Goossens et al. [24] also confirmed that the reproducibility was improved by adding the visual component. Thus, most of the studies on the respiratory signals using audio and/or visual devices during RT showed positive results with the device-assisted breathing compared to free breathing. The results of our study were shown to be in accord with these earlier ones with increasing reproducibility and regularity of respiratory amplitude and period using the complex system-guided breathing. However, the RMSE of breathing period (0.951 ± 0.130 sec, 0.741 ± 0.213 sec, and 0.464 ± 0.215 sec for free breathing, audio device-guided breathing, and complex system-guided breathing, resp.) was relatively big even for the complex system-guided breathing which might lead to change in phase and amplitude or movement of internal target. In addition, using the RPM system for the RGRT is possible on the assumption that the external motion is synchronized to the movement of the internal organs. Tsunashima et al. [25] analyzed the time difference of the respiratory signals and the three-dimensional tumor movement in 26 patients with lung, liver, and esophageal cancers. According to their report, the variation of the phase was within 0.3 seconds irrespective of organs, and the external signals were well correlated with the internal movement. The synchronism of the external and internal movement was not studied in the present study. Our future studies will follow these considerations. There are several demerits with present study such as a small patient number of 11 and no available data during the real treatment. However, the respiratory signals were collected from two different sessions for 5 minutes each for the three breathing conditions in 11 patients which meant about 1386 signals per the free, the audio-guided, and the complex system-guided breathing. In the latter problem, the actual treatment was done with the complex system-guided breathing and the signals of the free and the audio-guided breathing were not gathered in consideration of delay in the daily treatment time and increase in the workload of staff. Meanwhile, using the RPM system for the RGRT is possible on the assumption that the external motion is synchronized to the movement of the internal organs.

Meanwhile, according to Hau et al. [26], the use of RGRT with audio coaching was not associated with a significant reduction in spinal cord, esophagus, or cardiac dosimetric parameters. However, it did reduce the lung mean dose by 1.33 Gy (*P* < 0.001) and *V*_20 Gy_ by 2.2% (*P* < 0.001) with gating ranging 85–15% in patients with thoracic malignancies. Another study revealed that RGRT with DIBH technique correlated with 32% reduction (19.9% versus 13.5%) in lung *V*_20 Gy_ compared to free breathing in patients with esophageal cancer [17]. Our results showed the low dose volume areas of normal liver including *V*_5 Gy_, *V*_10 Gy_, and *V*_20 Gy_, and *V*_10 Gy_ received less doses of radiation with the 4D MIP CT than with the free breathing CT in patients with liver cancer. *V*_20 Gy_, *V*_30 Gy_, and *V*_40 Gy_ of ipsilateral lung with plans of 4D MIP CT were significantly favorable than that of the free breathing CT in patients with lung cancer. In spite of relatively wide range of gating (30–70%), the benefit of 4DRT with complex system-guided breathing compared to free breathing was quantified and showed statistical significance.

## 5. Conclusions

The reproducibility and regularity of respiratory amplitude and period were significantly improved with the complex system-guided breathing compared to free breathing and audio-guided breathing. In addition, the 30–70% gating 4DRT with the complex system-guided breathing was advantageous over free breathing in terms of DVH profiles of normal liver or lung.

## Figures and Tables

**Table 1:** The patients' characteristics.

Number	Age/sex	Diagnosis	Pathology	ECOG	Surgery	Concurrent chemotherapy	Breathing rate (/min)	Movement in fluoroscopy (cm)
1	65/F	HCC∗	HCC∗	1	Yes	No	12	2.5
2	64/M	HCC∗	HCC∗	1	Yes	No	15	3
3	56/F	HCC∗	HCC∗	1	No	No	18	1.8
4	65/M	HCC∗	HCC∗	0	No	No	17	1.5
5	62/M	HCC∗	HCC∗	0	No	No	16	1.2
6	52/M	HCC∗	HCC∗	0	No	No	10	1.8
7	59/M	NSCLC^†^	SCC^‡^	1	No	No	14	2.0
8	73/M	NSCLC^†^	SCC^‡^	2	No	No	12	1.2
9	80/M	NSCLC^†^	SCC^‡^	1	No	No	15	1.3
10	65/F	NSCLC^†^	AD^§^	0	No	No	13	1.5
11	62/M	NSCLC^†^	AD^§^	0	No	No	19	3.2

*Hepatocellular carcinoma.

^†^None-small-cell lung cancer.

^‡^Squamous cell carcinoma.

^§^Adenocarcinoma.

**Table:** (a) The RMSE of maxima (full inspiration)

	Free	Audio-guided	Complex system-guided	*P* value
Maxima (cm)	0.154 ± 0.096	0.119 ± 0.057	0.061 ± 0.024	0.031

Free breathing versus audio-guided breathing (*P* = 0.216).

Free breathing versus complex system-guided breathing (*P* = 0.004).

Audio-guided breathing versus complex system-guided breathing (*P* = 0.001).

**Table:** (b) The RMSE of minima (full expiration)

	Free	Audio-guided	Complex system-guided	*P* value
Minima (cm)	0.220 ± 0.085	0.174 ± 0.095	0.099 ± 0.053	0.007

Free breathing versus audio-guided breathing (*P* = 0.020).

Free breathing versus complex system-guided breathing (*P* = 0.000).

Audio-guided breathing versus complex system-guided breathing (*P* = 0.020).

**Table:** (c) The RMSE of period

	Free	Audio-guided	Complex system-guided	*P* value
Period (sec)	0.951 ± 0.130	0.741 ± 0.213	0.464 ± 0.215	0.046

Free breathing versus audio-guided breathing (*P* = 0.370).

Free breathing versus complex system-guided breathing (*P* = 0.009).

Audio-guided breathing versus complex system-guided breathing (*P* = 0.040).

**Table:** (a) Liver

	Free breathing CT plan (cc)	MIP based plan (cc)	*P* value
*V*_5 Gy_*	78.75 ± 3.51	68.06 ± 6.68	0.024
*V*_10 Gy_^†^	65.16 ± 7.83	54.24 ± 10.45	0.030
*V*_20 Gy_^‡^	43.84 ± 12.80	35.22 ± 14.11	0.048
*V*_30 Gy_^§^	32.81 ± 12.68	27.34 ± 14.68	0.117
*V*_40 Gy_^||^	25.79 ± 11.47	21.53 ± 13.61	0.069
*V*_50 Gy_^¶^	19.39 ± 9.71	16.06 ± 11.21	0.067

**Table:** (b) Ipsilateral lung

	Free breathing CT plan	MIP based plan	*P* value
*V*_5 Gy_*	48.97 ± 15.37	40.74 ± 11.07	0.051
*V*_10 Gy_^†^	37.71 ± 17.89	29.31 ± 15.22	0.035
*V*_20 Gy_^‡^	23.02 ± 11.20	16.03 ± 8.75	0.018
*V*_30 Gy_^§^	15.19 ± 9.24	8.94 ± 5.46	0.034
*V*_40 Gy_^||^	9.18 ± 5.88	4.90 ± 3.18	0.035
*V*_50 Gy_^¶^	1.64 ± 1.93	1.19 ± 1.35	0.287

**Table:** (c) Contralateral lung

	Free breathing CT plan	MIP based plan	*P* value
*V*_5 Gy_*	13.65 ± 12.29	8.91 ± 20.58	0.140
*V*_10 Gy_^†^	2.63 ± 4.90	1.01 ± 2.08	0.160
*V*_20 Gy_^‡^	0	0	—
*V*_30 Gy_^§^	0	0	—
*V*_40 Gy_^||^	0	0	—
*V*_50 Gy_^¶^	0	0	—

*Volume receiving 5 Gy.

^†^Volume receiving 10 Gy.

^‡^Volume receiving 20 Gy.

^§^Volume receiving 30 Gy.

^||^Volume receiving 40 Gy.

^¶^Volume receiving 50 Gy.
